# Ambient Conditions Prior to Tokyo 2020 Olympic and Paralympic Games: Considerations for Acclimation or Acclimatization Strategies

**DOI:** 10.3389/fphys.2019.00414

**Published:** 2019-04-24

**Authors:** Nicola Gerrett, Boris R. M. Kingma, Robert Sluijter, Hein A. M. Daanen

**Affiliations:** ^1^Department of Human Movement Sciences, Faculty of Behavioural and Movement Sciences, Amsterdam Movement Sciences, Vrije Universiteit Amsterdam, Amsterdam, Netherlands; ^2^TNO, The Netherlands Organization for Applied Sciences, Unit Defense, Safety and Security, Soesterberg, Netherlands; ^3^Royal Netherlands Meteorological Institute, De Bilt, Netherlands

**Keywords:** Olympics, thermal strain, WBGT, temperature, humidity, heat index, humidex

## Abstract

The Tokyo Olympics and Paralympic games in 2020 will be held in hot and humid conditions. Heat acclimation (in a climatic chamber) or heat acclimatization (natural environment) is essential to prepare the (endurance) athletes and reduce the performance loss associated with work in the heat. Based on the 1990–2018 hourly meteorological data of Tokyo and the derived wet bulb globe temperature (WBGT) (Liljegren method), Heat Index and Humidex, it is shown that the circumstances prior to the games are likely not sufficiently hot to fully adapt to the heat. For instance, the WBGT 2 weeks prior to the games at the hottest moment of the day (13:00 h) is 26.4 ± 2.9°C and 28.6 ± 2.8°C during the games. These values include correction for global warming. The daily variation in thermal strain indices during the Tokyo Olympics (WBGT varying by 4°C between the early morning and the early afternoon) implies that the time of day of the event has a considerable impact on heat strain. The Paralympics heat strain is about 1.5°C WBGT lower than the Olympics, but may still impose considerable heat strain since the Paralympic athletes often have a reduced ability to thermoregulate. It is therefore recommended to acclimate about 1 month prior to the Olympics under controlled conditions set to the worst-case Tokyo climate and re-acclimatize in Japan or surroundings just prior to the Olympics.

## Introduction

The Tokyo 2020 Olympics are expected to be the hottest ever with daily air temperatures exceeding 30°C and wet bulb globe temperature (WBGT) (>28 during the hottest part of the day), exceeding that of previous games (Kakamu et al., 2017). These conditions expose athletes to extremely challenging conditions in which one cannot perform optimally. It is undisputed that heat acclimation or acclimatization, hereafter collectively referred to as HA, brings about physiological adaptations in humans to lessen the thermal strain as a result of enhanced thermoregulatory capabilities. And since precooling opportunities are limited in most sports (Bongers et al., 2017), the athletes strongly depend on HA to prepare for the environmental conditions. Athletes and their support teams have two options for HA protocols: acclimation or acclimatization. Acclimation is the physiological or behavioral changes that reduces the strain or enhances the endurance of the strain brought about by artificial exposure to the climatic conditions (IUPS Thermal Commission, 2001). This requires the use of climatic chambers, which are expensive, require trained professionals and are not widely accessible; questioning the feasibility of this protocol for all athletes. Alternatively, they can adopt an acclimatization protocol whereby physiological or behavioral changes that reduces the strain arise from exposure to a natural climate (IUPS Thermal Commission, 2001). Namely, athletes may travel to Japan prior to the Olympics and adapt to the local climate naturally but the ambient conditions prior to the Olympic games may not reflect the actual conditions expected during the games. Both techniques have advantages and disadvantages and which protocol to adopt should be a key consideration for athletes and their support teams.

The reported physiological adaptations to HA protocols include a lower resting and exercising core body temperature (T_c_), a lower heart rate, a higher sweat rate (SR) alongside a diluted sweated ion concentration, expanded plasma volume and a lower T_c_ for the onset of sweat production and vasodilation (Sawka et al., 2001). The physiological adaptations that occur serve to attenuate the thermal strain and can also improve athletic performance in warm-hot conditions (Lorenzo et al., 2010; Garrett et al., 2012; Racinais et al., 2015). Périard et al. (2015) summarized the time course of various physiological adaptations; suggesting that the adaptation process begins with the first exposure and complete HA occurs after approximately 14 days. Full adaptation for reduced HR, core and skin temperatures taking about 7 days and the longest requirement for full adaptation is about 2 weeks for SR responses. This information is generally based on studies that utilized acclimation protocols and only a few studies used heat acclimatization. A recent study showed that athletes from countries with a warm climate performed better than athletes from countries with a cold climate during the Marathon des Sables (Ioannou et al., 2018). To our knowledge no study has directly compared heat acclimation with heat acclimatization. Although there is some evidence that tolerance to higher T_c_ is greater in those acclimatized over several weeks compared to those acclimated to 1–2 weeks (Sawka et al., 2001). However, the difficulty in making direct comparisons is substantial. It remains unclear which technique offers the best advantage for complete HA.

For athletes traveling far, jet lag, travel fatigue and adjustments of circadian rhythms also must be considered. In this sense it may be appealing to travel to Japan several weeks prior to the games in order to adjust circadian rhythms and heat adapt to the local climate using heat acclimatization. However, there is a considerable risk that the local climate prior to the games is not stressful enough to elicit the necessary adaptations. The Tokyo 2020 Olympics are scheduled 24th July until 9th August with the month of August having the highest ambient temperature conditions. The Paralympics are scheduled from August 25th to September 6th. Japan has distinct seasons so it is highly probable that the environmental temperatures in June and July are lower than that expected during the games. For optimal heat adaptation, it is recommended that the environmental conditions (i.e., temperature and humidity) should be similar to, or higher than that expected during competition (Taylor et al., 1997). Additionally, there is some evidence that the physiological adaptations for rectal temperature and heart rate are not evident when sufficient recovery is not available during a HA protocol (Daanen et al., 2011). To ensure optimal heat adaptations, the conditions expected during the Tokyo 2020 Olympics games should ideally be utilized during a HA protocol with relatively low thermal strain after the heat exposure to allow for sufficient recovery. The aim of this paper is to describe the ambient conditions prior to and during the Olympic and Paralympic games based on meteorological data from the past 28 years that include the effects of global warming. Using this information, we can describe the conditions expected during the games and assess the risk of incomplete adaptations through heat acclimatization.

## Materials and Methods

Meteorological data were derived from the Japan Meteorological Agency. Data were collected specifically from Tokyo ward (i.e., the city) of Tokyo prefecture; where most of the events will take place and includes the location of the Olympic village and the Olympic stadium. Some events are scheduled in neighboring prefectures to Tokyo such Kanagawa (sailing), Shizuoka (cycling), Ibaraki (football), Yokohama (football, baseball, and softball), and Saitama (football, basketball, and golf) which have similar environmental conditions to Tokyo. Some football events are also scheduled for prefectures north of Tokyo in Hokkaido (Sapporo; 830 km), Miyagi (Rifu; 310 km), and Fukushima (Fukushima; 240 km) prefecture, with the former having considerable cooler environmental conditions than Tokyo prefecture. For succinctness, the data presented only include meteorological data from Tokyo ward (Tokyo prefecture).

Hourly data were collected from the period of June 1 to September 6 (final day of the Paralympics) from 1990 to 2018. Meteorological data is available from 1989 but due to inconsistent gaps in data sampling during the year of 1989 we decided to exclude this year from our analysis. The data collected included temperature (°C), dew point temperature (°C), relative humidity (%), solar radiation (MJ/m^2^), and wind speed (m/s).

To determine the ambient conditions in Tokyo on the lead up to and during the Olympic and Paralympic games we calculated the number of days prior to the start of the Tokyo 2020 games. For example, June 1st is identified as day -53; being 53 days before the start of the Olympics. July 24th is the official start date and as such is coded as 0 and ends on August 9th, coded as Day 16. The Paralympics are scheduled from August 25th until September 6th which are coded as Day 32 to Day 44. These data were plotted alongside single meteorological data (temperature and relative humidity) and biometeorological indices [Heat Index (HI), Humidity Index (Humidex) and the WBGT].

### Meteorological and Biometeorological Indices

Acclimation protocols are conducted in controlled climatic conditions based on temperature and relative humidity and thus these were included in our analysis. Biometeorological indices are generally better predictors of thermal strain than single meteorological variables and thus we used the meteorological data to calculate the following: Humidex, HI and WBGT [see the following papers for more detailed descriptions of these indices (Budd, 2008; Blazejczyk et al., 2012)]. Humidex uses air temperature and air vapor pressure to reflect the perceived temperature (Ho et al., 2016) and was calculated using the following equation:

Humidex=T+0.5555⋅(vp−10)

Where,

$$vp=6.11\cdot e^{\left(5.417.753\cdot \left(\frac{1}{273.16}\right)\cdot \left(\frac{1}{273.16+td}\right)\right)}$$

Where, *td* is dew point temperature (in °C) and *e* is Euler’s number.

The Humidex values are categorized as: 20–29 = *no discomfort*, 30–39 = *some discomfort*, 40–45 = *great discomfort*; *avoid exertion*, and ≥46 = *Dangerous*; *possible heat stroke*.

Heat Index is somewhat similar in that it as it combines air temperature and relative humidity to determine an apparent temperature, which indicates how hot it feels (Rothfusz, 1990). HI was calculated using the following equation:

$$\begin{array}{ll} HI= & -42.379+2.04901523\cdot T+10.14333127\cdot RH\\ & -0.22475541\cdot T\cdot RH\\ & -0.00683783\cdot T\cdot T-0.05481717\cdot RH\cdot RH\\ & +0.00122874\cdot T\cdot T\\ & \cdot RH+0.00085282\cdot T\cdot RH\cdot RH-0.00000199\cdot T\cdot T\cdot \\ & RH\cdot RH \end{array}$$

Where, *RH* is relative humidity (%) and *T* is temperature (°F). The temperature data from the Japan Meteorological Agency was converted from °C into °F to calculate the *HI*.

The output value from the HI calculation is categorized with the following descriptors: 27–32 = *Caution* (fatigue is possible with prolonged exposure and/or physical activity), 32–41 = *Extreme Caution* (sunstroke, muscle cramps, and/or heat exhaustion possible with prolonged exposure and/or physical activity), 41–54 = *Danger* (sunstroke, muscle cramps, and/or heat exhaustion likely, heat stroke possible with prolonged exposure and/or physical activity), ≥54 *Extreme Danger* (heat stroke or sunstroke likely).

As Japanese summers are known to be especially hot and humid, the Humidex and the HI were considered appropriate for inclusion and are common to the United States and Canada, respectively. WBGT was also chosen as it is more internationally recognized and used by many sporting organizations and thus familiarity is greater. WBGT was calculated using the following equation:

WBGT(°C)=0.7⋅wetbulb+0.2⋅Tglobe+0.1.Tdrybulb

Where, *wetbulb* is the wet-bulb temperature, *Tglobe* is the globe temperature and *Tdrybulb* is the dry bulb temperature.

WBGT was calculated from meteorological data using the Liljegren method (Liljegren et al., 2008) using the Heat stress package in R developed by Meteo Suisse^1^. Data for solar radiation collected from the Japan Meteorological Agency was measured in MJ/m^2^/hr and converted by a factor of 0.0036 to W/m^2^.

Different sports governing bodies define the recommendations for activities at various WBGT ranges. As a general summary, the following recommendations are provided for continuous activity and competition for WBGT <18.3°C is “Generally safe/normal activity”; 18.4–22.2°C “risk of exertional heat stroke and heat illness; high risk individuals should be monitored or not compete”; 22.3–25.6°C “risk for all competitors increases”; 25.7–27.8°C “risk for unfit, non-acclimatized individuals is high”; 27.9–30.0°C “cancel level for exertional heat stroke risk”; 30.1–32.2°C “cancel or stop practice and competition”; ≥32.3°C “Cancel exercise” (based on ASCM position stand, Armstrong et al., 2007). The category ranges and the associated risks are reduced in those who are fit, low risk and acclimatized individuals engaging in training or non-continuous activity.

The time schedule for Tokyo 2020 Olympics had not been announced at the time of writing this paper, therefore we assumed a time span of 08:00–21:00 h based on the three previous Olympics (Rio, London and Beijing), thus excluding night time temperatures. Data were averaged during this time period. To determine the worst-case scenario, we also averaged the data between 12:00 and 15:00 h. Data are also plotted for every hour of the day, averaged from day 0–16 and from day 32–44 for the start and end of the Olympics and Paralympics, respectively.

The average values over the 1990–2018 periods may not reflect the actual situation during the Tokyo 2020 Olympics due to numerous factors such as year-to-year variations, urban heat island effect and climate change. Using the Hothaps database (Kjellstrom et al., 2009, 2014) we extrapolated the yearly changes in WBGT, HI, and Humidex values to the year 2020 to correct for global warming. Hothaps monitors the effects of climate change worldwide and estimates the increase in meteorological and biometeorological indices per decade. The data generated were specific to Tokyo prefecture. Temperature, dew point temperature, and WBGT are estimated to have the following yearly increases: 0.038, 0.009, and 0.025°C, respectively. The collected data are based over 28 years with 2004 as the central year. Taking 2004 as the central year we then applied a correction of 0.61°C for temperature, 0.14°C for dew point temperature and 0.40°C for WBGT and applied this to the collected data (average of the 1990–2018 period) to get the best estimate for the Tokyo 2020 Olympics. The temperature increase during the 1990–2018 period includes the urban heat island effect. The population in Tokyo prefecture increased from approximately 11.9 million in 1990 to 13.2 million in 2010^2^. Assuming a similar exponential growth, the number of inhabitants is predicted to increase to 15.2 million in 2020. If we take the urban heat island effect (2.01 log P – 4.06) (Oke, 1973), this results in 0.16°C increase in ambient temperature of the city center from the 1990–2018 average to 2020. Thus, the linear extrapolation of 0.61°C in temperature may slightly underestimate the real warming due to non-linear growth of the Tokyo population.

The probability of WBGT on the days prior to the Olympic games being similar to the expected conditions during the Olympic games for different time periods (08:00–11:00, 12:00–15:00, and 16:00–19:00 h) were calculated. To generate probability curves, we used the 50th and 95th percentile of the WBGT of the specific day prior to the Olympics and compared this to the actual percentiles during the Olympic period. In this way we avoided the skewness (-1.05) of the WBGT data during the Olympic period. Although some skewness exists in the data (from day 10 onward) the variation for the parameters (temperature, relative humidity, HI, Humidex, and WBGT) is expressed as standard deviation.

## Results

### 24-h Meteorological and Biometeorological Indices During the Olympics

**FIGURE 1:**
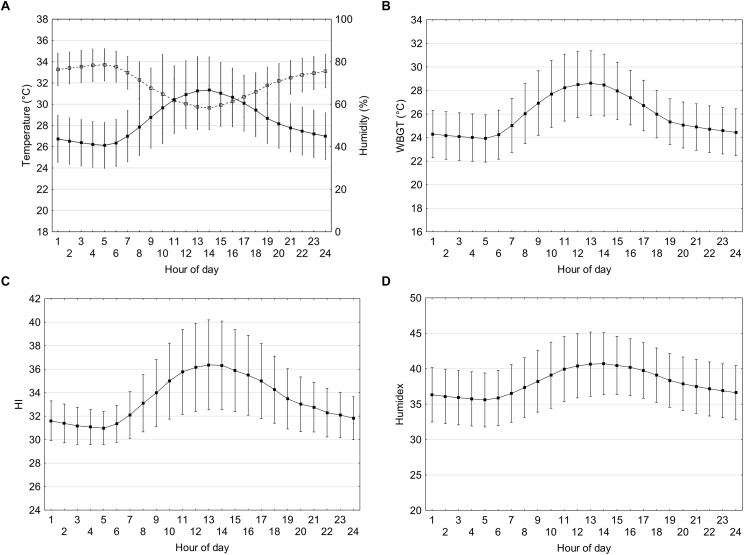
Temperature (black squares) and relative humidity (open squares) **(A)**, WBGT **(B)**, HI **(C)**, and Humidex **(D)** at hourly intervals based on meteorological data from the past 29 years (1990–2018) during the dates corresponding to the Olympic period (July 24th until August 9th). Vertical bars represent one standard deviation.

Hourly ambient temperature, relative humidity, WBGT, HI, and Humidex for the Olympic period (24th July until 9th August) are shown in Figure 1A–D. Solar radiation was slightly skewed so descriptive data (minimum, median, maximum, first, and third quartile) are shown in Table 1. The ambient temperature is lowest during the night time at approximately 05:00 h and begins to rise from 07:00 h, peaking to 31.3 ± 3.1°C at 14:00 h. As expected the relative humidity peaks when the ambient temperature is lowest around 05:00 h at 79 ± 8% and as temperature rises the relative humidity declines to 58 ± 10% at the hottest part of the day and rises thereafter.

**Table 1:** Minimum, first quartile, median, third quartile, and maximum solar radiation at hourly intervals based on meteorological data from the past 29 years (1990–2018) during the dates corresponding to the Olympic period (July 24th until August 9th) and Paralympic period (August 25th until September 6th).

	Solar radiation (W/m^2^) Olympic period					Solar radiation (W/m^2^) Paralympic period				
	Minimum	First quartile	Median	Third quartile	Maximum	Minimum	First quartile	Median	Third quartile	Maximum
05:00	0	0	0	3	14	0	0	3	6	22
06:00	0	19	42	69	122	0	9	39	72	158
07:00	0	58	131	206	328	0	26	106	192	411
08:00	0	111	233	392	553	3	49	194	358	569
09:00	8	153	353	539	711	3	68	286	506	733
10:00	0	194	483	678	853	3	85	331	619	869
11:00	0	236	561	783	942	4	95	408	703	961
12:00	0	286	636	839	953	2	115	450	728	1000
13:00	0	281	608	825	942	3	109	456	747	981
14:00	0	242	550	764	900	3	99	419	689	914
15:00	0	225	469	650	797	3	82	367	592	817
16:00	0	153	331	489	614	2	60	253	419	631
17:00	0	97	189	306	422	0	34	150	264	444
18:00	0	42	86	139	225	0	15	69	122	217
19:00	0	8	17	25	67	0	3	14	25	47
20:00	0	0	0	0	3	0	0	0	0	3
21:00–04:00	0	0	0	0	0	0	0	0	0	0

The WBGT is lowest during the night time at approximately 05:00 h (23.9 ± 2.0) and begins to rise from 06:00 h, peaking to 28.6 ± 2.8°C at 13:00 h. The Humidex is lowest during the night time at approximately 05:00 h (35.6 ± 3.8) and begins to rise from 06:00 h, peaking to 40.6 ± 4.5 at 13:00 h. HI also follows a similar pattern, with the lowest during the night time at approximately 05:00 h (31.0 ± 1.4) and begins to rise from 06:00 h, peaking to 36.4 ± 3.8 at 13:00 h. Solar radiation on a cloudless sky peaks at 917 W/m^2^ at the hottest part of the day (12:00 h).

### 24-h Meteorological and Biometeorological Indices During the Paralympics

**FIGURE 2:**
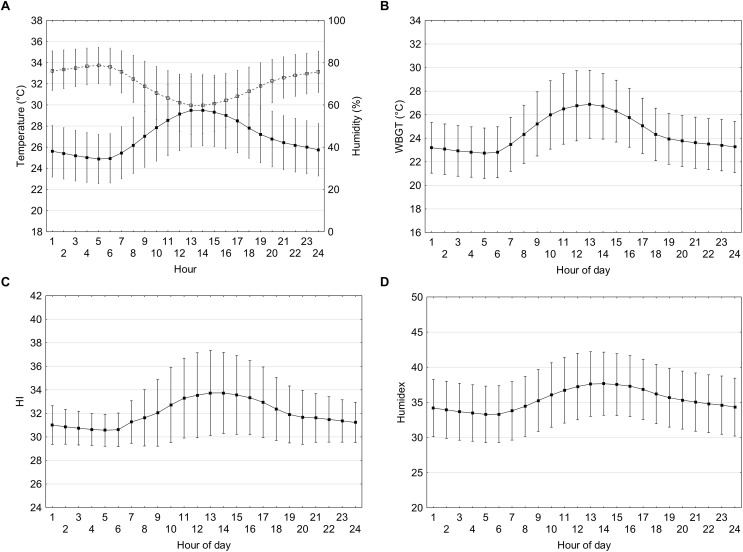
Temperature (black squares) and relative humidity (open squares) **(A)**, WBGT **(B)**, HI **(C)**, and Humidex **(D)** at hourly intervals based on meteorological data from the past 29 years (1990–2018) during the dates corresponding to the Paralympic period (August 25th until September 6th). Vertical bars represent one standard deviation.

Hourly ambient temperature, relative humidity, WBGT, Humidex, and HI during the Paralympic period (August 25th to September 6th) are shown in Figure 2A–D and solar radiation in Table 1. The ambient temperature is lowest (25.0 ± 2.3°C) during the night time at approximately 05:00 h and begins to rise from 07:00 h, peaking to 29.7 ± 3.4°C at 13:00 h. Relative humidity peaks when the ambient temperature is lowest around 05:00 h at 78 ± 9% and as temperature rises the relative humidity declines to 59 ± 13% at the hottest part of the day and rises thereafter.

The WBGT is lowest during the night time at approximately 05:00 h (22.8 ± 2.2°C) and begins to rise from 06:00 h, peaking to 27.0 ± 2.9 at 13:00 h. The Humidex is lowest during the night time at approximately 05:00 h (33.5 ± 4.1) and begins to rise from 06:00 h, peaking to 37.8 ± 4.6 at 13:00 h. HI also follows a similar pattern, with the lowest during the night time at approximately 05:00 h (30.6 ± 1.4) and begins to rise from 06:00 h, peaking to 33.4 ± 3.5 at 13:00 h. Solar radiation on a cloudless sky peaks at 1000 W/m^2^ at the hottest part of the day (12:00 h).

### Meteorological and Biometeorological Indices Prior to and During the Olympics and Paralympics (08:00–21:00 h)

**FIGURE 3:**
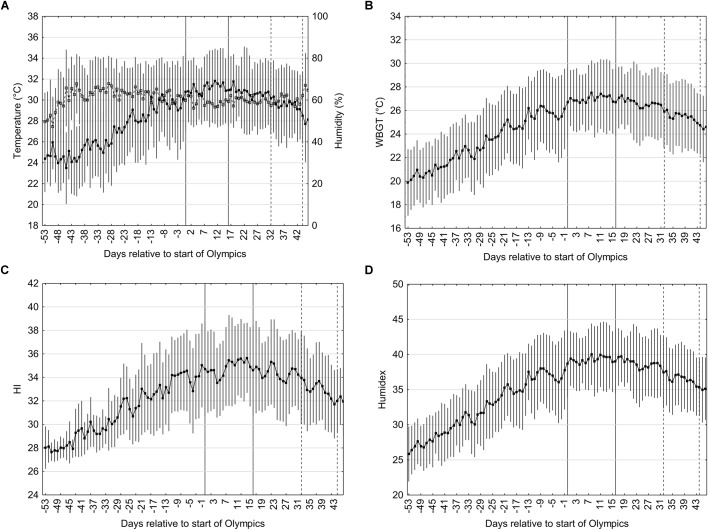
Temperature (black squares) and relative humidity (open squares) **(A)**, WBGT **(B)**, HI **(C)**, Humidex **(D)** on the days prior to the start of the Olympics and Paralympics. Data is the mean (±SD) during the time period of 0800–2100 h obtained from hourly metrological data from the past 29 years (1990–2018). The Olympics period is coded on day 0 to 16 and the Paralympics on day 32 and 44; the former is in between the solid lines and the latter between the dotted lines. Vertical bars represent one standard deviation.

Based on previous Olympics games, the typical competing period was between 08:00 and 21:00 h. The ambient temperature, relative humidity, WBGT, Humidex, and HI on the days prior to the Olympics during this time period are shown in Figure 3A–D. This data is based on hourly data averaged between 08:00 and 21: 00 h when it is expected that the games will take place. Fifty-three days prior to the Olympics the ambient temperature is 22.9 ± 3.1°C and continues to rise to a plateau during the Olympics period, after which temperature starts to drop. Relative humidity reaches its peaks and plateaus much more quickly (by day -40) and remains high throughout the Olympic and Paralympic period. For the Olympic period the average temperature and relative humidity is 29.7 ± 3.1°C and 65 ± 11%. The Paralympic period has an average temperature of 28.2 ± 3.2°C and relative humidity of 65 ± 13%.

The WBGT at day -53 is 19.9 ± 2.8°C and rises steadily until day -1 followed by a plateau until day 17. The average WBGT during the Olympic period is 27.0 ± 2.8°C. After this period, the WBGT slowly starts to decline. The average WBGT for the Paralympics is 25.4 ± 3.0°C. Humidex at day -53 is 25.9 ± 3.9 and rises steadily until day 2 followed by a short plateau until day 19 and slowly declines thereafter. The average Humidex during the Olympic period is 39.3 ± 4.3 and 36.5 ± 4.5 during the Paralympic. HI at day -53 is 28.8 ± 1.8 and rises steadily until day 3 followed by a short plateau until day 15 and thereafter declines. The average HI during the Olympic and Paralympic periods are 34.8 ± 3.4 and 33.0 ± 3.3, respectively.

### Meteorological and Biometeorological Indices Prior to and During the Olympics and Paralympics (12:00–15:00 h)

**FIGURE 4:**
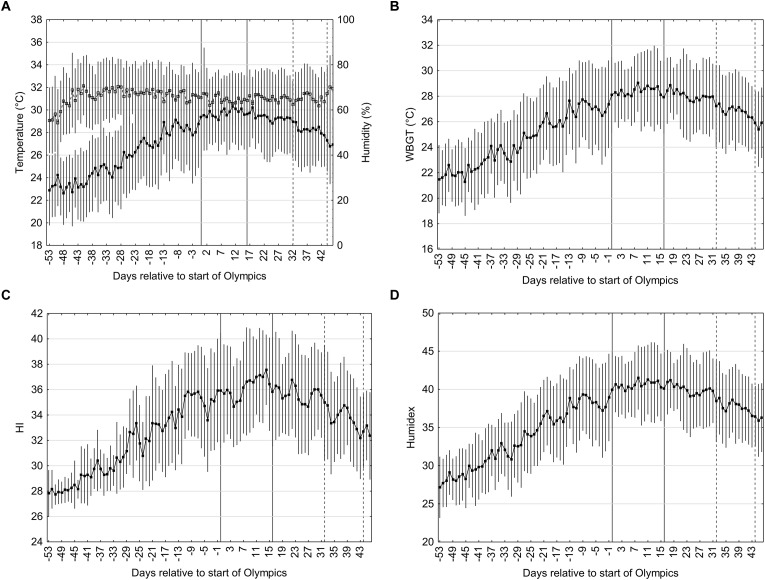
Temperature (black squares) and relative humidity (open squares) **(A)**, WBGT **(B)**, HI **(C)**, Humidex **(D)** on the days prior to the start of the Olympics and Paralympics. Data is the mean (±SD) during the time period of 12:00–15:00 h obtained from hourly metrological data from the past 29 years (1990–2018). The Olympics period is coded on day 0 to 16 and the Paralympics on day 32 and 44; the former is in between the solid lines and the latter between the dotted lines. Vertical bars represent one standard deviation.

The hottest period of the day is between 12:00 and 15:00 h and may represent the worst-case scenario during the Olympic and Paralympic games. The ambient temperature, relative humidity, WBGT, Humidex, and HI on the days prior to the Olympics during this time period are shown in Figure 4A–D.

The ambient temperature rises from 24.3 ± 3.2°C on day -53 until approximately day -3 where the temperature peaks and plateaus, declining slowly from day 17 onward. The relative humidity rises from 50 ± 14% on day -53 and rises to a long plateau on day -38 with small variations during the Olympic and Paralympic period. The temperature and RH during the Olympic period between 12:00 and 15:00 h are 31.3 ± 3.1°C and 59 ± 10%. The temperature and RH during the Paralympic period are 29.5 ± 3.4°C and 60 ± 13%.

The WBGT at day -53 is 21.5 ± 2.7°C and rises steadily until day 7 followed by a plateau until day 17, declining thereafter. The average WBGT during the Olympic and Paralympics periods are 28.4 ± 2.8°C (5th and 95th percentile: 23.1 and 31.7, skewness -1.05) and 26.8 ± 3.0°C (5th and 95th percentile; 21.6 and 30.6, skewness -0.4). Humidex at day -53 is 27.2 ± 4.0 and rises steadily until day -1 followed by a short plateau until day 15, declining thereafter. The average Humidex during the Olympics and Paralympics are 40.7 ± 4.4 and 37.8 ± 4.5, respectively. HI at day -53 is 27.8 ± 1.8 and rises steadily peaking at day 11, declining thereafter. The average HI during the Olympics and Paralympics are 36.1 ± 3.7 and 33.8 ± 3.6, respectively.

### Chance of Successful Acclimatization

**FIGURE 5:**
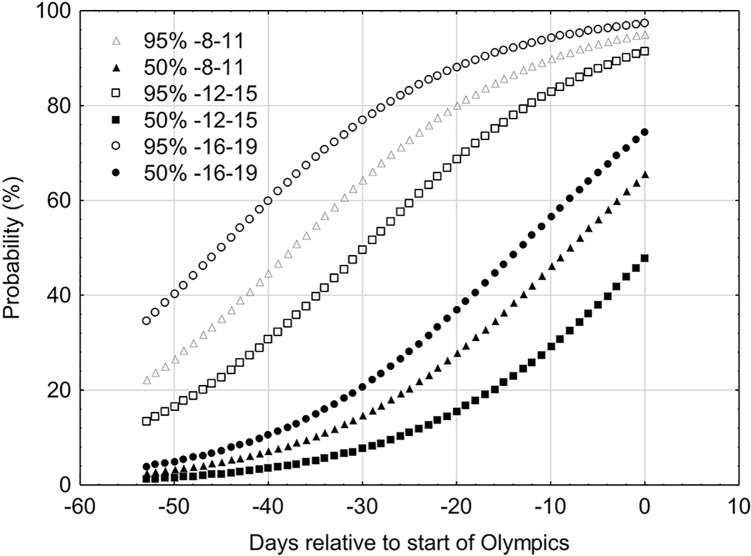
The probability of WBGT (50th and 95th percentile) being similar to the Tokyo 2020 Olympics on the days preceding the games at the following time periods: 08:00–11:00, 12:00–15:00, and 16:00–19:00 h.

The probability of WBGT on the days prior to the Olympic games being similar to the expected conditions during the Olympic games were calculated and plotted in Figure 5. Using the 50th percentile for 08:00–11:00, 12:00–15:00, and 16:00–19:00 h, generated the following respective logistic equations:

$$\begin{array}{l} probability\;(\%)=\frac{1}{1+e^{-0.08}(0-8.1)}\\ probability\;(\%)=\frac{1}{1+e^{-0.08}(0-1.1)}\\ probability\;(\%)=\frac{1}{1+e^{-0.08}(0-13.3)} \end{array}$$

Where, *e* is Euler’s number.

Using these equations, it can be calculated that 14 days prior to the Olympic games at 08:00–11:00 h there is a 38%, chance of WBGT being similar to that expected during the Olympic games. Between 12:00–15:00 and 16:00–19:00 h the chance of similar conditions during the Olympic games are 23 and 49%, respectively.

To account for the maximum temperatures (i.e., the 95th percentile), the curve shifts upward and to the left. Thus, 14 days prior to the Olympics there is 87% chance of the expected WBGT occurring at 08:00–11:00 h and 78% at 12:00–15:00 h. The chance of the conditions being similar to those during the games between 16:00 and 19:00 h is 92%. The probability curves for the 95th percentile for 08:00–11:00, 12:00–15:00, and 16:00–19:00 h generated the following respective logistic equations:

$$\begin{array}{l} probability\;(\%)=\frac{1}{1+e^{-0.08}(0-37.4)}\\ probability\;(\%)=\frac{1}{1+e^{-0.08}(0-29.8)}\\ probability\;(\%)=\frac{1}{1+e^{-0.08}(0-45.1)} \end{array}$$

## Discussion

The first purpose of this study was to describe the ambient conditions during the Tokyo 2020 Olympic and Paralympic games based on meteorological data from the past 28 years in Tokyo city corrected for global warming. Hourly data for ambient temperature and relative humidity and three indices of heat strain (WBGT, Humidex, and HI) were calculated for both the Olympic and Paralympic periods. Using this data, we can describe the expected conditions for the upcoming games and highlight average and peak conditions. Furthermore, the second purpose of this study was to describe the ambient conditions on the days prior to the start of the Olympic games and assess the risk of incomplete adaptations through heat acclimatization. Athletes, coaching staff and sports governing bodies will find the descriptive data presented useful for knowing the expected environmental conditions and for designing optimal preparation strategies.

### Hourly Meteorological and Biometeorological Indices During the Games

The average estimated WBGT values during the Tokyo 2020 Olympics games are 27.0 ± 2.8°C averaged over the day and 28.4 ± 2.8°C over the hottest period of the day (Figure 3, 4, respectively). The latter values are in the 27.9–30°C “cancel level for exertional heat stroke risk” range for continuous activity/competition which may be relevant for endurance based events such as the marathon (Armstrong et al., 2007). According to the ACSM guidelines (Armstrong et al., 2007) for non-continuous activity, which may be more relevant for team based intermittent events, the risk to the athlete is lower when heat acclimatized and events canceled when the WBGT exceeds 32.3°C. Kakamu et al. (2017) calculated that on average 49 days in Japan exceed the 27.9°C threshold. However, this may be an overestimation as the WBGT estimation of Kakamu et al. (2017) was based on the Australian Bureau of Meteorology (ABM) model and may show considerable deviations from the real WBGT in particular in cloudy situations (Grundstein and Cooper, 2018). We used a method that has been shown to estimate WBGT from meteorological data with greater precision (Liljegren et al., 2008). Given that the variation in the data is large the chances of the WBGT exceeding 27.9 threshold is high (85%), whilst the chances for exceeding the threshold for 32.3°C are only 15% for the hottest part of the day (WBGT 28.4 ± 2.7°C).

During the Olympic games the average daily Humidex and HI are 39.3 ± 4.3 and 34.8 ± 3.4, respectively. If athletes are competing during the hottest period of the day (12:00–15:00 h) the Humidex and HI values of 40.7 ± 4.4 and 36.1 ± 3.7 could be expected. According to the guidelines for Humidex this would expose individuals to “some discomfort” during most of the day but “great discomfort” during the hottest periods, with the recommendation to “avoid exertion.” For HI this would expose athletes to conditions categorized as “Extreme caution” with sunstroke, muscle cramps and/or heat exhaustion possible with prolonged exposure and/or physical activity. The heat strain indices, Humidex and HI, specify “danger” levels at values ≥46 and 41–54, respectively. Figure 1C,D shows that whilst these levels may not be attained during the hottest part of the day (Humidex; 40.7 ± 4.4 and HI; 36.1 ± 3.7), the standard deviations are considerable so there is a possibility that those conditions could be exceeded during the Olympics and Paralympics. Kosaka et al. (2018) measured the ambient conditions along the course of the marathon route during July and August, with either 06:30 h or 07:30 h start times. The best- and worst-case scenario for temperature and relative humidity were 26.3 ± 0.85°C and 51.8 ± 3.4% and 34.3 ± 1.8°C and 47.4 ± 5.6%, respectively. Furthermore, no cloud cover alongside high ambient temperature and relative humidity during the hottest period of the day (see Table 1) can add considerable thermal challenges and negate performance (Otani et al., 2019). The worst-case scenario during the Olympics could be solar radiation of 855–917 W/m^2^ between 12:00 and 15:00 h. The evidence to date suggests that athletes and coaches may consider preparing for the worst-case scenario; heat adaptation strategies, pre- and per-cooling, clothing, reducing time outdoors, and strategies to promote behavioral thermoregulation should be considered.

The Paralympic games are scheduled August 25th until September 6th; 16 days after the Olympics whereby the ambient temperatures have begun to decline. Nevertheless, the average daily temperature and relative humidity during the Paralympic games are 28.2 ± 3.2°C and 65.0 ± 12.7% and can be expected to reach 29.5 ± 3.4°C and 59.6 ± 13.0% during the hottest periods of the day. Solar radiation could be as high as 914–1000 W/m^2^ during the hottest period of the day when there is minimal cloud cover. These conditions still impose a considerable thermal challenge and even more so as some Paralympic athletes have impaired thermoregulatory functions (Price, 2006; Griggs et al., 2015). There is less evidence for the application of heat strain indices in special populations and sporting events for disabled athletes (Girard, 2015). Guidelines are available from some sports governing bodies for para-athletes employing similar or lower thresholds for the suspension of play compared to able-bodied athletes. The International Tennis Federation for example suspends play when WBGT ≥28°C. During the Paralympic period the estimated daily WBGT is 25.4 ± 3.0 and 26.8 ± 3.0°C during the hottest periods of the day. Given the variation in the data the possibility of conditions exceeding this threshold is likely. The average daily HI and Humidex are 33.0 ± 3.3 and 36.5 ± 4.5, respectively. If athletes are competing during the hottest period of the day (12:00–15:00 h) the Humidex and HI values of 37.8 ± 4.5 and 33.8 ± 3.6 could be expected. For Humidex “some discomfort” would be expected and the HI would vary between “caution” to “extreme caution.” However, we could not find any information about whether these categories are appropriate for special populations. Given the diverse health conditions of Paralympic athletes and the elevated risk of heat related illness amongst those with impaired thermoregulatory functions (e.g., spinal cord injured individuals) the thermal challenge still exists and should be prepared for.

### Heat Acclimate or Heat Acclimatize?

Based on the expected environmental conditions described previously it is evident that the conditions will impose thermal challenges and all athletes are advised to take additional precautions to ensure safe and optimal performances. A highly effective method to prepare for the environmental conditions can be achieved through heat acclimation or heat acclimatization protocols. The former involves creating an artificial environment usually in a climatic chamber and exposing oneself to similar conditions expected during the games for a given period of time. This can typically be achieved over 5–14 days with approximate daily exposures of 2 h/day. To magnify the thermal stress, Taylor et al. (1997) recommended using a dry bulb temperature at least equivalent to the highest anticipated and where possible elevate this a further 5–10°C; with 40°C as the upper limit for humid conditions (>60% RH). Based on these recommendations and the reported meteorological data the environmental conditions employed in an acclimation protocol should be >31 and ≤40°C with >60% RH. Recommended WBGT values to use for optimal HA are not widely reported but based on the expected WBGT values during the Olympic games a WBGT ≥28.4°C should be considered. Recently there has been less focus on the environmental conditions in which these acclimation sessions are completed but rather using ambient conditions sufficient enough to raise core temperature >38.5°C quickly and keeping it elevated for 60 min (Garrett et al., 2012; Gibson et al., 2015; Neal et al., 2016). Whilst this technique of controlled hyperthermia focuses on the core temperature responses rather than the ambient conditions, conditions which restrict heat loss (i.e., hot and humid) will result in a faster elevation in core temperature and require a lower metabolic heat production to maintain an elevated core temperature >38.5°C. The physiological adaptations that accompany controlled hyperthermia are reported to occur more quickly and more completely than standard HA protocols (Taylor et al., 1997; Périard et al., 2015; Daanen et al., 2018). However, heat acclimatization has been favored in the literature for inducing physiological and psychological adaptations specific to the conditions where the event will take place (Hellon et al., 1956; Edholm, 1966; Périard et al., 2015; Racinais et al., 2015). Previous studies have shown an improved thermal tolerance in acclimatized compared to acclimated individuals, although direct comparisons are difficult as conditions were not similar between groups (Edholm, 1966; Sawka et al., 2001; Ioannou et al., 2018). Furthermore, the impact of solar radiations and air flow are often unaccounted for in acclimation protocols as they are difficult to replicate in climatic chambers but they have a large impact on overall heat stress in the field (Saunders et al., 2005). If the controlled hyperthermia protocol is used for HA, it has to be realized that it requires considerable exercise intensities to achieve the target core temperatures if the athlete chooses to acclimatize in the Tokyo climate about 2–3 weeks prior to the Olympics. The WBGT 2 weeks prior to the games at the hottest moment of the day (13:00 h) is only 26.4 ± 2.9°C compared to 28.6 ± 2.8°C during the games. The high exercise intensity may interfere with the tapering protocol that is generally used to recover from extensive exercise in the period prior to the Olympics (Mujika and Padilla, 2003).

Traveling to Japan several days to weeks prior to the Olympic games to acclimatize naturally to the environmental conditions is an attractive, feasible and alternative strategy to acclimation. It may be particularly advantageous when access to climatic chambers are not possible and also provides an opportunity for the adjustment of circadian rhythms if traveling far to Japan. The heat strain indices (temperature, WBGT, Humidex, and HI) are all considerably lower prior to the Olympics. The indices rise on the days leading up to the Olympics; peaking and plateauing during the Olympic games. Under these circumstances the thermal stress prior to the Olympics may be too low to achieve optimal adaptations (Taylor et al., 1997) or lead to exercise intensities interfering with tapering. The advice to use an acclimatization strategy may only be applicable if one is certain of stable and similar conditions prior to the event itself. Assuming that the environmental conditions required for successful acclimatization are equal to, or above, the conditions expected during competition (Taylor et al., 1997). Figure 5 provides some indication of whether full acclimatization can occur. A 14-day HA strategy is often suggested for full adaptations, in similar environmental conditions (Taylor et al., 1997; Périard et al., 2015). If an athletes’ scheduled competition is between 12:00 and 15:00 h and they employ a 14-day acclimatization strategy in Tokyo at the same time of day then at the start of heat acclimatization there is a 23% chance (50th percentile) of the conditions between similar to the Olympic games. This means that there is a strong risk (77% chance) of the WBGT being less than that expected during the Olympic games. Throughout the 14-day acclimatization protocol this chance reduces to 53% of the WBGT being less than that expected during the Olympic games. If we consider the 95th percentile, i.e., as an indicator of the maximum heat strain during that day, then the chances during a 14-day acclimatization protocol of WBGT being less than the Olympics ranges from 8 to 22%. Schasfoort (2014) compared the meteorological conditions 2–3 weeks prior to and during the 1996, 2000, 2004, and 2008 Olympics and 12 other major sports events. Thermal stress was lower during the 2000 and 2004 Olympics prior to the games, but not for 1996 and 2008. In particular the 2012 UEFA European Soccer Championships in Ukraine (maximum temperature prior: 21.4°C, during: 26.3°C) and the 2011 Men’s Hockey Champions Trophy in India (prior: 25.1°C; during 29.6°C) showed significantly lower thermal stress prior to than during the games. The data indicates that there is some risk of incomplete adaptation if an acclimatization protocol in Tokyo is employed.

Figure 5 provides useful information to the organizing committee of the Olympic games. The probability of conditions reaching the 50th percentile for WBGT by day 0 is only 48% for events scheduled between 12:00–15:00 and 66 and 75% for events scheduled between 8:00–11:00 and 16:00–19:00 h. Such information may be pertinent for the organization committee of the Olympics to schedule high risk heat stress events, such as the marathon, toward the end of the Olympic games to allow athletes more opportunity to acclimatize naturally. When the event schedules are announced, Figure 1–5 will provide useful advice on the ideal times to acclimatize to the heat. It is expected that prolonged and sustained high intensity efforts will be held early in the morning (<09:00 h) where the WBGT is <26°C (Figure 1B). Figure 4B shows that if 2 weeks prior to the Olympic games, the average WBGT is >26°C 2 weeks prior to the Olympic games. Based on our calculations if athletes choose to travel to Japan and acclimatize naturally during the 2 weeks preceding the games then the chances of WBGT being >26°C averages 67% from 12:00 to 15:00 h. Alternatively, when events are scheduled during the hottest periods (12:00–15:00) during the Olympics then sub optimal acclimatization conditions should be expected due to insufficient heat stress in the weeks prior to the event. In such situation’s athletes/coaching staff may consider supportive strategies to facilitate heat acclimatization. Post-exercise hot water immersion has recently showed some positive results to facilitate physiological adaptations to heat (Zurawlew et al., 2016). As long as core body temperature is monitored to ensure athletes are working within safe boundaries then overdressing during training may also be administered (Ely et al., 2018; Willmott et al., 2018).

Studies show the physiological and performance benefits of training in the heat for competition in temperate conditions (Shvartz et al., 1979; Lorenzo et al., 2010; Corbett et al., 2014). To the authors knowledge there are no acclimation studies employing environmental conditions lower than those used during a heat stress test. Indeed, a review based on approximately twenty-one heat acclimation studies indicated that the conditions used for acclimation were the same or higher than those imposed during a heat stress test (Daanen et al., 2018). Karlsen et al. (2015) did, however, utilize natural acclimatization (34 ± 3°C, 18 ± 5%) and tested performance in conditions that were, perhaps by chance rather than design, lower than the conditions used during a heat stress test in a climatic chamber (44 ± 3°C, 44 ± 5%). Performance during a cycling time trial relative to performance in an unacclimatized state, improved by 11 ± 8 and 5 ± 4% after 6 and 13 days (respectively) of natural acclimatization. Without a comparison it is difficult to determine whether HA in environmental conditions below the expected competition conditions provides a sufficient stimulus to negate detriments to performance in the heat; more importantly, whether the adaptations are optimal. This uncertainty elevates the risk of incomplete adaptations if an acclimatization strategy in Japan is used. Paralympic athletes on the other hand stem to benefit from the higher ambient conditions prior to the Paralympic games. For the hottest parts of the day the WBGT is 26.8 ± 3.0 and 28.4 ± 2.8°C during the Paralympic and Olympics, respectively. It may be feasible to acclimatize in Japan prior to the Paralympic games but athletes and coaches should fully consider the safety implications of training in the heat especially for those with impaired thermoregulatory abilities that results from their impairment and/or medication.

An important challenge is incorporating the high physical demands of a HA protocol within an athletes busy training schedule. The effects of HA begin to decay very quickly, with most physiological adaptations generally disappearing in 28 days (Williams et al., 1967; Armstrong and Maresh, 1991; Daanen et al., 2018). It is therefore advisable to HA as close as possible to competition or to maintain the acclimation status using several short periods of heat re-acclimation (Casadio et al., 2017; Daanen et al., 2018). There is some research to suggest that following a decay, the time period for regaining the previously attained physiological adaptations is reduced (Givoni and Goldman, 1973; Pandolf et al., 1977; Weller et al., 2007; Ashley et al., 2015) and one study even found that supercompensation existed in the physiological adaptations (Saat et al., 2005). Thus, it is possible to complete a full HA protocol 1 month prior to competition and then complete a shorter re-HA protocol just prior to competition. Within the context of Tokyo 2020 Olympics, traveling to Japan several days-to-weeks to acclimatize naturally to the environment imposes a risk of incomplete adaptations as the data presented clearly indicates lower ambient conditions prior to the Olympic games. If athletes have access to a climatic chamber and can complete a controlled hyperthermia acclimation strategy then this may allow athletes to acclimate fully prior to the games. Athletes can then incorporate their tapering phases within the decay period. They can travel to Japan with sufficient time for circadian adjustments and re-acclimatize in Japan, where the chances of the conditions being similar to competition are greater.

### Limitations

We aimed to estimate the ambient conditions in Tokyo city (Tokyo prefecture) during the 2020 Olympic games based on metrological data from the past 28 years. Climate forecasting is difficult and our analyses are estimations. The variation in the data may be larger than we present, especially as climate change has resulted in more extreme weather occurrences and challenges future forecasting. We aim to provide an estimation that considers the urban heat island effect, Tokyo’s growing population and climate change but there are numerous factors uncontrolled for within our estimation.

To summarize, we provided an overview of the predicted environmental conditions prior to and during the Tokyo 2020 Olympic and Paralympic games. It is evident that there is a considerable thermal challenge and athletes and coaching staff are advised to prepare for these conditions. Whilst acclimatization is a feasible strategy, data are presented to assess the probability of the conditions prior to the Olympics being lower than during the Olympics. This information can be extremely useful in the planning and designing of strategies to allow the athletes to best prepare for the environmental conditions of the Tokyo 2020 Olympic and Paralympics games.

## Author Contributions

NG, RS, BK, and HD were involved in the conceptual ideas, data collection, analysis, interpretation, and manuscript presentation.

## Conflict of Interest Statement

The authors declare that the research was conducted in the absence of any commercial or financial relationships that could be construed as a potential conflict of interest.

## Footnotes

1 https://rdrr.io/github/anacv/HeatStress/

2 https://www.e-stat.go.jp/en
